# ﻿Seven *Epithemia* taxa (Bacillariophyta) from Lake Akan (Japan) and their salinity tolerances

**DOI:** 10.3897/phytokeys.229.104449

**Published:** 2023-07-18

**Authors:** Takashi Chiba, Yoshifumi Horie, Akihiro Tuji

**Affiliations:** 1 College of Agriculture, Food and Environment Sciences, Department of Environmental and Symbiotic Science, Rakuno Gakuen University, Bunkyodai 582, Midorimachi, Ebetsu, Hokkaido 069-8501, Japan Rakuno Gakuen University Ebetsu Japan; 2 Research Center for Inland Seas (KURCIS), Kobe University, Fukaeminami-machi, Higashinada-ku, Kobe, Hyogo 658-0022, Japan Kobe University Kobe Japan; 3 Department of Botany, National Museum of Nature and Sciences, Tsukuba, Ibaraki 305-0005, Japan Department of Botany, National Museum of Nature and Science Tsukuba Japan

**Keywords:** Diatom, *Epithemia* genus, Lake Akan, salinity

## Abstract

The ecologies (salinity tolerance) of many diatoms are largely unknown, despite their potential to contribute to more detailed paleoenvironmental reconstructions. This study therefore aimed to investigate the relationship between diatom species and salinity. We cultured seven cosmopolitan benthic diatom species obtained from Lake Akan, a freshwater inland lake in Japan: *Epithemiaadnata*, *E.frickei*, *E.gibba*, *E.operculata*, *E.sorex*, *E.* sp. and *E.turgida*. Each species was cultured at eleven salinities between 0‰ and 50‰. *Epithemiaadnata*, *E.frickei* and *E.sorex* had the highest growth rate at a salinity of 3‰, with no further increase observed above 25‰. However, *E.gibba* had the highest growth rate at a salinity of 5‰, with no increase at salinities ≥ 30‰. These results suggest that *E.adnata*, *E.frickei*, *E.gibba*, and *E.sorex* grow in freshwater to brackish-water environments. *Epithemiaoperculata* and *E.* sp. proliferated at all salinities, indicating that they can adapt to hypersaline environments. However, *E.turgida* did not survive in salinities >10‰, making it the species with the narrowest salinity tolerance range. These results provide new knowledge that improves the understanding of the ecology of these species in modern environments and offer insights into paleoenvironmental reconstructions through diatom analysis.

## ﻿Introduction

**Figure 1.**
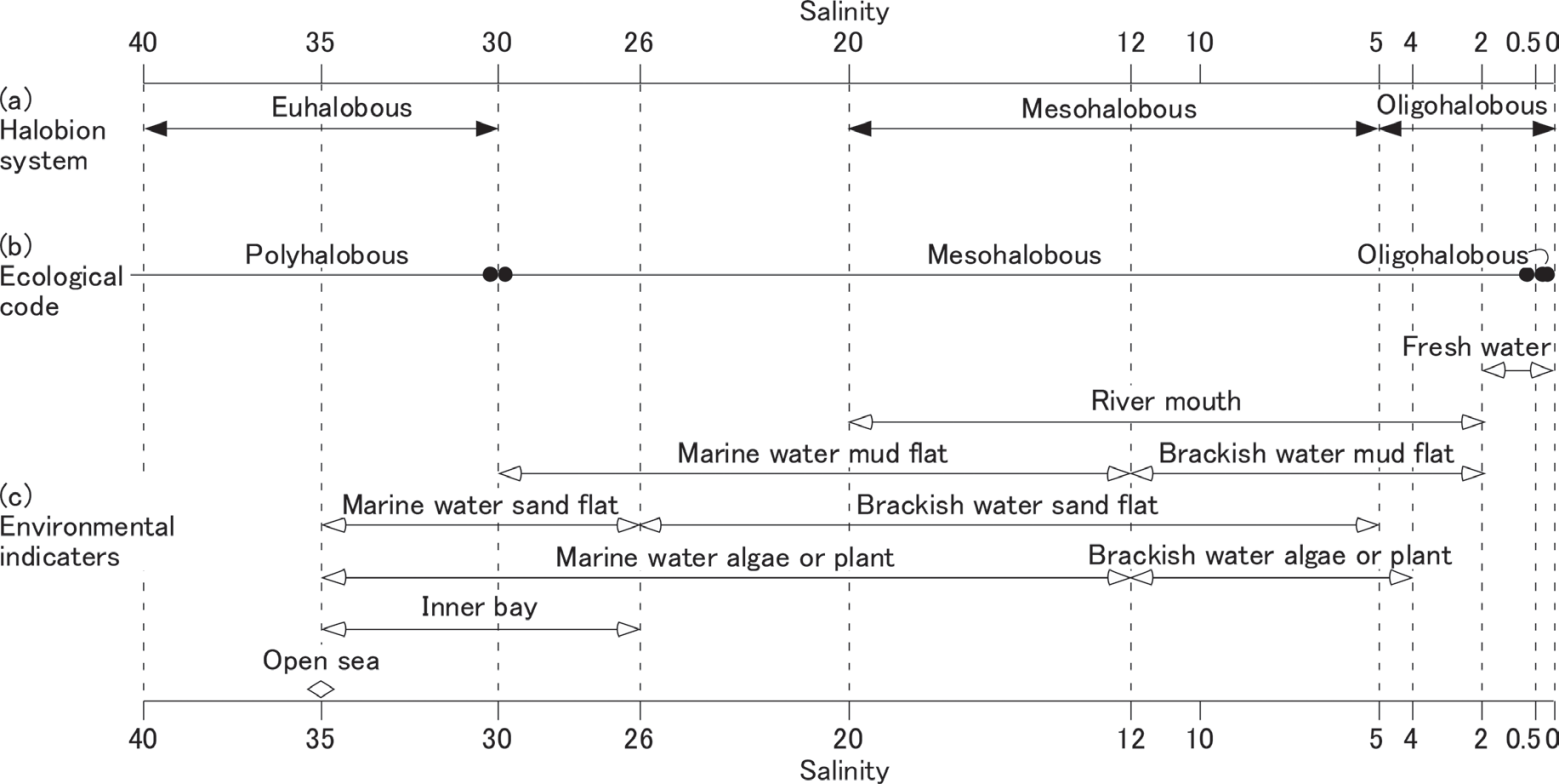
The correspondence of salinity ranges between among halobion system, ecological code and environmental indicators. **a** shows salinity ranges of halobion system (Kolbe 1927). Polyhalobous, mesohalobous and oligohalobous correspond to marine, brackish-water and freshwater respectively **b** shows salinity ranges of ecological code (Vos and De Wolf 1988) **c** shows salinity ranges of ten diatom assemblages in environmental indicators (Kosugi 1988) (modified from Chiba and Sawai 2014).

Diatoms are unicellular algae that are used as environmental indicators because of their adaptive radiation through the aquatic environment (Vos and De Wolf 1993; Van Dam et al. 1994). They are used as indicators in modern and paleoenvironmental reconstructions of sedimentary environments because their siliceous (SiO_2_·nH_2_O; amorphous silica) frustules are well preserved in sediments, and they appear as fossil diatoms (Kolbe 1927; Vos and De Wolf 1988; Chiba and Sawai 2014) (Fig. 1). Diatoms are particularly useful indicators for reconstructions of coastal environmental changes, such as changes in paleo-sea level (Smol and Stoermer 2010). However, many diatom species still have unknown ecologies (Round et al. 1990); despite their abundance in Holocene sediments, it is unclear whether they are freshwater or brackish-water species. Although it is important to determine this characteristic in brackish-water environments, it can be challenging to determine through fieldwork alone. However, culture experiments are an effective method for clarifying these uncertainties. (Smayda 1969; Yamamoto et al. 2017).

Owing to the difficulty of taxonomy, the genus *Epithemia*, a taxon within raphid diatoms (Bacillariophyta), has been continuously described as a new species because of its high diversity and numerous framework recombinations (You et al. 2009; Stancheva et al. 2013; Vishnyakov et al. 2014; Ruck et al. 2016; Rybak et al. 2020). Moreover, the information accumulated for this genus is underutilised in paleoenvironmental reconstructions. On the other hand, the genus *Epithemia* in current taxonomy is widely adapted and distributed from freshwater to marine environments.

For example, *Epithemiagibba* (Ehrenberg) Kützing, formerly known as *Rhopalodiagibba* (Ehrenberg) O. Müller, is a cosmopolitan benthic species (epipelon species on sand grains or aquatic plants) commonly found in lakes, rivers, and coastal regions worldwide; however, it prefers waters with relatively high electrolyte concentrations (Patrick and Reimer 1975) and alkalinity (Van Dam et al. 1994). Ruck et al. (2016) proposed taxonomic changes in Rhopalodiales and Surirellales based on molecular phylogenetic analysis. Rhopalodiaceae was integrated with Surirellaceae and the paraphyletic genus *Rhopalodia* was integrated with *Epithemia* (Kamakura and Sato 2018; Kamakura et al. 2021). However, in reconstructions of paleocoastal environments from Holocene sediments, *E.gibba* is sometimes classified as a freshwater species (Zalat and Vildary 2007; Tsoy et al. 2015), although it often appears in sediments and is sometimes classified as a brackish-water species (Chiba et al. 2016; Nazarova et al. 2022). Understanding the ecology (salinity range) of this species would make it important in paleoenvironmental reconstructions; however, detailed knowledge of its salinity tolerance, as well as those of *E.adnata* (Kützing) Brébisson and *E.sorex* Kützing (El Hamouti and Gibert 2012; Roy and Keshri 2015; Chiba et al. 2016) is lacking, despite their potential to provide more detailed paleoenvironmental reconstructions.

In this study, therefore, we conducted culture experiments using seven *Epithemia* species isolated from the water of Lake Akan, an inland lake in eastern Hokkaido, Japan, and investigated in detail the relationships between their growth rates and salinity.

## ﻿Materials and methods

### ﻿Samples

**Figure 2.**
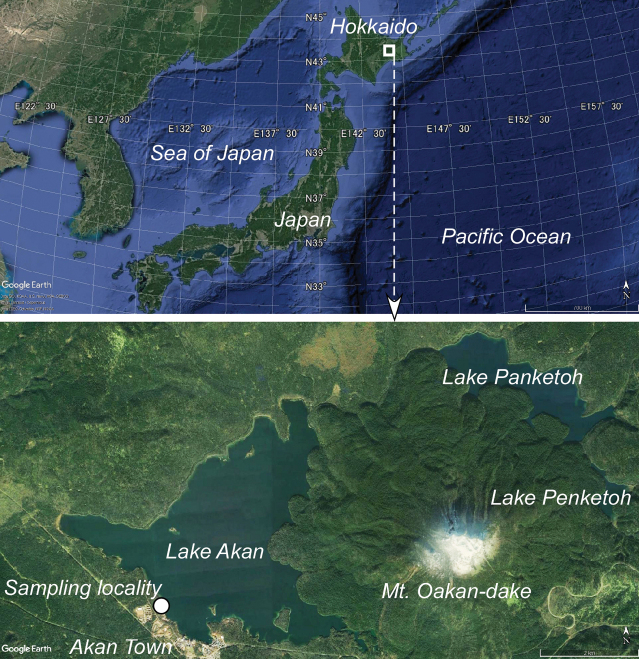
Sampling locality.

Samples were obtained in July 2022 from the shore of Lake Akan (Fig. 2; 43°26′25.80″N, 144°5′6.70″E), eastern Hokkaido, Japan, using 10-mL syringes. Lake Akan is an inland volcanic lake that serves as a habitat for rare algae such as *Aegagropilalinnaei* Kützing (e.g., Wakana et al. 2021). It has a salinity, pH, and temperature of 0‰, 8.0, and 21 °C, respectively (Table 1), with an electrical conductivity of 366 (μS/cm) and sand as the bottom sediment. Kawashima and Mayama (2002) previously reported several species of the genus *Epithemia* among six taxa from Lake Akan.

**Table 1.** Water quality in the sampling locality.

	GPS coordinates	Temperature (°C)	Salinity (‰)	Electric conductivity (μS/cm)	pH
Sampling locality	43°26'25.80"N, 144°5'6.70"E	21	0	366	8.0

### ﻿Isolation and culture

Vegetative cells of *E.adnata*, *E.frickei*, *E.gibba*, *E.operculata*, *E.sorex*, *E.* sp. and *E.turgida* were isolated from samples using Pasteur pipettes and an inverted microscope (CK-2, Olympus, Tokyo, Japan). The cells of these seven species were attached to aquatic plants and sand-size grains in Lake Akan, so they are considered epiphytic and benthic species, respectively. Cells of each species were transferred into 11 wells of a 12-well culture plate (VTC-P12, AS ONE CORPORATION, Osaka, Japan) to establish strains. The salinities in the wells were set to 0‰, 1‰, 3‰, 5‰, 10‰, 15‰, 20‰, 25‰, 30‰, 35‰, and 50‰, according to Yamamoto et al. (2017). Many wells were set to salinities ≥10‰ because, although these species were reported from freshwater, we wanted to test the upper limits of their salinity tolerances. The pH of all wells was set to 8.3 (weakly alkaline). The culture medium was based on Bold’s Basal Medium (BBM) (Stein 1973), and its concentration was adjusted by adding artificial seawater and silica. The cultures were maintained in an incubator at 15 °C under 12-h light:12-h dark conditions.

### ﻿Experimental protocol

The experimental protocol followed that of Tuji (2000). Each well of the 12-well cultures plates that we used had an area of 3.8 cm^2^ and depth of 2 cm. Medium (1 mL) was pipetted into each well of a 12-well plate prepared in triplicate for each salinity, and pre-incubated strains exhibiting logarithmic growth were inoculated into each well at a starting density of one cell per well (Yamamoto et al. 2017). The long-term culture experiments were initiated on 15 July 2022 without a salinity acclimation period, and the number of diatoms was counted at least once every seven days after inoculation until growth stopped and stationary phase was confirmed. The experimental period varied for each strain but did not exceed 60 d, with all experiments completed by 15 September 2022.

The specific growth rates during the exponential growth period were calculated using the following equation (Shimizu 2006):

µ[d^−1^] = Log_e_(*N*/*N*_0_)/(*t* − *t*_0_)

where µ is the growth rate, *t*_0_ and *t* are the initial and final days of the exponential growth period, respectively, and *N*_0_ and *N* are the cell numbers at *t*_0_ and *t*, respectively. After completing all experiments, the strains were boiled in H_2_O_2_ to remove organic material, washed, and mounted on permanent slides. The frustules were examined using an optical microscope (BH-2, Olympus), and a scanning electron microscope (JSM-6390LV, JEOL Ltd., Tokyo, Japan) was used to identify the species. Morphological analysis of *Epithemia* species was conducted according to Krammer and Lange-Bertalot (1988), Kawashima and Mayama (2002), and You et al. (2009). Some *Epithemiaadnata* (Kützing) Brébisson varieties are indistinguishable from *E.frickei* Krammer. Therefore, we selected those varieties that could be morphologically distinguished by observation with an inverted microscope and used them for the experiment.

## ﻿Results

In this section, we first show the morphological characteristics of isolated and cultured species (Figs 3–9) and describe their salinity tolerance (Fig. 10).

### ﻿*Epithemiaadnata* (Kützing) Brébisson

**Figure 3.**
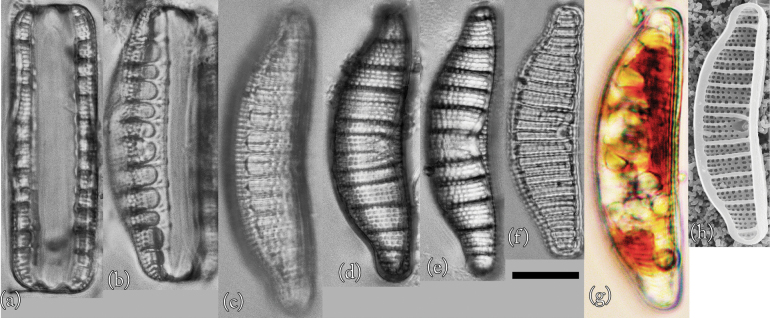
LM (**a–g**) and SEM (**h**) images of *Epithemiaadnata*. Scale bar: 10 μm.

This species is reported by Kawashima and Mayama (2002). Taxonomic examination confirmed that this species is *Epithemiaadnata* (Fig. 3). Individuals observed in culture had universal morphological characteristics recognized worldwide (Krammer and Lange-Bertalot 1988). However, many individuals were close to the smallest size reported thus far.

**Light microscopy (LM) morphology.** Valves were approximately centred on the dorsum, the dorsal margin was slightly convex, the ventral margin was slightly concave or straight, and the apex was broad and rounded (Krammer and Lange-Bertalot 1988). Individuals were 24–52 μm in length and 7–10 μm in width (*n* = 50). The raphe was biarcuate; that is, bent inwards from the pole towards the dorsal side, but never reaching its edge. There were 12–17 areola in 10 μm, 13–17 striae in 10 μm, and 4–6 costae in 10 μm, with 3–6 striae between adjacent costae. The costae were nearly parallel or slightly radial.

**Scanning electron microscopy (SEM) morphology.** The external raphe was surrounded on both sides by thin silica strips, forming a V-shaped structure in the middle of the ventral margin (Krammer and Lange-Bertalot 1988). In the external valve view, a regular and uniform arrangement of dome-shaped caps was connected at the apical and transapical ends. These dome-shaped caps were usually four to eight in number and formed a single areola. The very complex structure of these areolas made their identities and boundaries more complex (Krammer and Lange-Bertalot 1988). The raphe fissure was located approximately the same distance from both edges of the apical region. A hyaline band was present on the dorsal side of the fissure and ran along the length of the valve (Krammer and Lange-Bertalot 1988).

**Proliferative salinity.** This species grew at all salinities from 0‰ to 50‰. The growth rate was highest at a salinity of 3‰, and there was almost no growth at salinities of 25‰ or higher (Fig. 10).

### ﻿*Epithemiafrickei* Krammer

**Figure 4.**
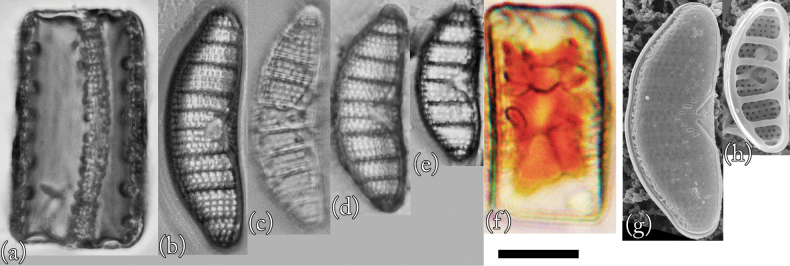
LM (**a–f**) and SEM (**g** and **h**) images of *Epithemiafrickei*. Scale bar: 10 μm.

This is the first record of this species in Lake Akan. Morphological examination confirmed that this species is *Epithemiafrickei* (Fig. 4). Individuals observed in culture had universal morphological characteristics recognized worldwide (Krammer and Lange-Bertalot 1988). However, many individuals were close to the relatively smaller size reported to date.

**LM morphology.** Morphological features included many characteristics similar to those of *E.adnata*, including dorsoventral flaps, a slightly convex dorsal margin, and a slightly concave ventral margin. However, apices were slightly rounded and very slightly protruding. Individuals were 15–28 μm in length and 7–10 μm in width (*n* = 50). The raphe was essentially double arced; that is, bent inwards from the bar towards the dorsal side, but not reaching its end. There were 11–16 areola in 10 μm, 13–17 striae in 10 μm, and 4–6 costae in 10 μm, with 3–6 striae between adjacent costae. The costae were almost parallel or slightly radial.

This species resembles *E.adnata*, but it is smaller. In addition, the areola density is coarse. Furthermore, the overhang of the valve end is not as large as that of *E.adnata*, and it converges. Individuals with frustules similar in length to those of *E.adnata* tended to have wider frustules. These features can be identified by LM.

**SEM morphology.** The overhang on the frustule end of this species was much smaller than that of *E.adnata* and converged. In addition, the shapes of areolae were more random than those of *E.adnata*. The external raphe was bounded on both sides by thin bands of silica, and the raphe formed a V-shaped structure in the centre of the ventral margin (Krammer and Lange-Bertalot 1988; Kawashima and Mayama 2002). The raphe fissure was located approximately the same distance from both edges of the apical region. (Krammer and Lange-Bertalot 1988; Kawashima and Mayama 2002). These characteristics are similar to those of *E.adnata*. However, the sequence interval of areolae within one of the striae lines of this species tended to be slightly wider than those of *E.adnata*.

**Proliferative salinity.** This species grew at all salinities from 0‰ to 50‰. The growth rate was highest at a salinity of 3‰, and there was almost no growth of this species at salinities of 25‰ or higher. This characteristic is similar to that of *E.adnata*.

### ﻿*Epithemiagibba* (Ehrenberg) Kützing

**Figure 5.**
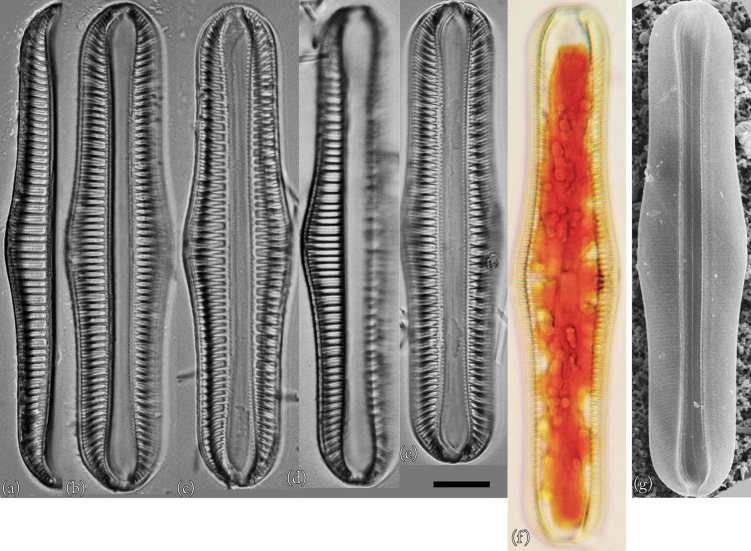
LM (**a–f**) and SEM (**g**) images of *Epithemiagibba*. Scale bar: 10 μm.

This species is reported as *Rhopalodiagibba* by Kawashima and Mayama (2002). Morphological examination confirmed that this species is *Epithemiagibba* (Fig. 5). Individuals observed in culture had universal morphological characteristics recognized worldwide (Krammer and Lange-Bertalot 1988). However, many individuals were also close to the smallest size reported to date (Krammer and Lange-Bertalot 1988; Kawashima and Mayama 2002).

**LM morphology.** Valves were linear or bracket-shaped, and apices were bent towards the ventral margin (Krammer and Lange-Bertalot 1988; You et al. 2009). The central valve was inflated, and it was slightly indented towards the ventral margin at the central nodule. Individuals were 74–96 µm in length and 7–9 µm in width (*n* = 50). The raphe was biarcuate; that is, branches curved from the poles inwards towards the dorsal side, but they never reached the margin. There were 14–17 areolae in 10 µm, 13–17 striae in 10 µm, and 7–9 costae in 10 µm, with 2–3 striae between adjacent costae. Costae were near-parallel or radiated slightly.

**SEM morphology.** Some individuals had spherical silica deposits on the surface of the valves, whereas others had none. Areolae were usually indistinct (Kawashima and Mayama 2002), but some had bands arranged parallel to the valves, and some had bands that were depressed inwards.

**Proliferative salinity.** This species grew at all salinities from 0‰ to 50‰. The growth rate was highest at 5‰, and there was almost no growth of this species at salinities of 30‰ or higher (Fig. 10).

### ﻿*Epithemiaoperculata* (C. Agardh) Ruck & Nakov

**Figure 6.**
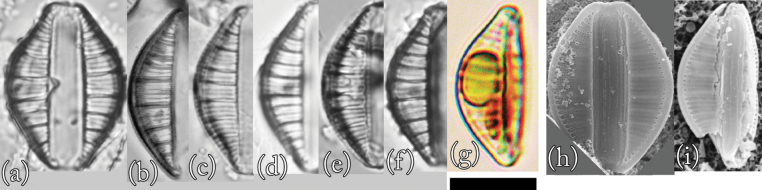
LM (**a–g**) and SEM (**h** and **i**) images of *Epithemiaoperculata*. Scale bar: 10 μm.

This is the first record of this species in Lake Akan. Taxonomic examination confirmed that this species is *Epithemiaoperculata* (Fig. 6).

**LM morphology.** Frustules were elliptical and close to triangular. The dorsal edge of the valves was arched, but the central part was slightly depressed ventrally. The ventral margin was arcuate at the apices and straight in the axial direction at the centre. The apices of the valves extended and protruded. Individuals were 12–18 µm in length and 6–8 µm in width (*n* = 50). The raphe was biarcuate. There were 13–16 striae in 10 µm and 6–8 costae in 10 µm, with 2 striae between adjacent costae. Costae were nearly parallel or radiated slightly. There were 5–7 costae in 10 µm. This species is similar to *Epithemiarupestris* (W. Smith) Krammer and *Epithemiaconstricta* W. Smith, as shown by Lange-Bertalot and Krammer (1987) and Krammer and Lange-Bertalot (1988). Although it was difficult to distinguish between small individuals of this species and *E.operculata* by optical microscopic observation, it was possible to distinguish them based on their different curvatures.

**SEM morphology.** There were 36–44 areolae in 10 µm, and double puncta formed the striae (Watanabe et al. 2005; You et al. 2009). The keel was raised above the valve plane and slightly indented towards the ventral margin at the central nodule (You et al. 2009).

**Proliferative salinity.** This species grew at all salinities tested in this study. The growth rate was the fastest at a salinity of 50‰, but no clear peak was observed (Fig. 10).

### ﻿*Epithemiasorex* Kützing

**Figure 7.**
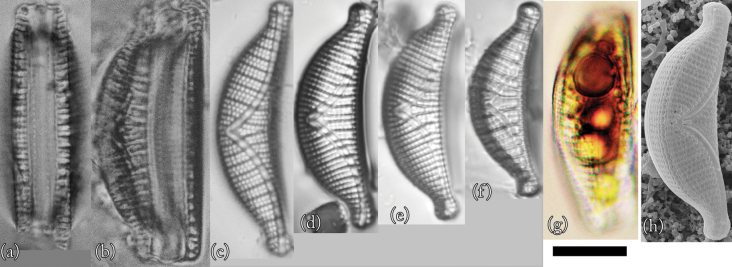
LM (**a–g**) and SEM (**h**) images of *Epithemiasorex*. Scale bar: 10 μm.

This species is reported by Kawashima and Mayama (2002). Taxonomic examination confirmed that this species is *Epithemiasorex* (Fig. 7). Individuals observed in culture had universal morphological characteristics recognized worldwide (Krammer and Lange-Bertalot 1988). Many individuals, however, were close to the smallest size reported to date (Krammer and Lange-Bertalot 1988; Kawashima and Mayama 2002).

**LM morphology.** Frustules were approximately dorsoventral, the dorsal margin was slightly convex, and the ventral margin was slightly concave. The apices of the frustules were thin and rounded. Individuals were 24–32 μm in length and 7–9 μm in width (*n* = 50). The raphe was biarcuate; this is, bent inwards from the pole towards the dorsal side, but not reaching its edge. There were 14–17 areola in 10 μm, 13–16 striae in 10 μm, and 6–9 costae in 10 μm. Frustules had 2–3 striae between adjacent costae, and costae were almost parallel or slightly radial.

**SEM morphology.** A wide hyaline band was located dorsal to the raphe fissure. The acroraphe fissure was relatively simple and located in the middle of the pole (You et al. 2009). The external view of the valves showed an arrangement of solid domed caps positioned above the areolae (You et al. 2009).

**Proliferative salinity.** The growth rate was highest at a salinity of 5‰, and there was almost no growth of this species at salinities of 25‰ or higher (Fig. 10).

### ﻿*Epithemia* sp.

**Figure 8.**
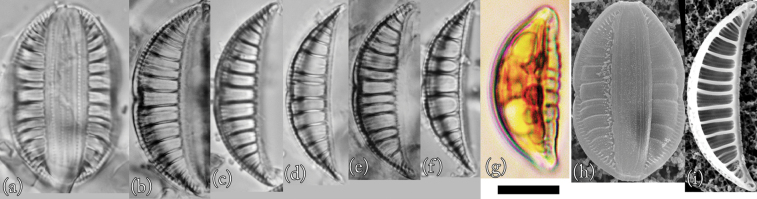
LM (**a–g**) and SEM (**h** and **i**) images of *Epithemia* sp. Scale bar: 10 μm.

This is the first description of this species in Lake Akan. Taxonomic examination confirmed that this species is *Epithemia* sp. (Fig. 8).

**LM morphology.** The frustules were broadly elliptical or arcuate and crescent-shaped. The dorsal edge of the valves was arched, but the central part was depressed ventrally. However, the ventral margin was arcuate at the apices and straight in the axial direction at the centre. The apices of the valves extended in an arc and protruded. Individuals were 28–35 µm in length and 6–8 µm in width (*n* = 50). The raphe was biarcuate. There were 4–7 costae in 10 µm. Costae were nearly parallel or radiated slightly. This species is similar to *Epithemiarupestris* (W. Smith) Krammer, as shown by Krammer and Lange-Bertalot (1988). This species is also similar to *Epithemiarumrichiae* (Krammer) Krammer (as *Rhopalodiarumrichiae*; Ohtsuka and Tuji 1999), but the vesicle density was clearly finer. Although it was difficult to distinguish small individuals of this species from those of *E.operculata* by optical microscopic observation, it was possible to distinguish them by their different curvatures.

**SEM morphology.** External observation using SEM showed the central part of the valve exhibiting areola and constriction of the central portion. The keel was raised above the valve plane and clearly indented towards the ventral margin at the central nodule. Striae formed two areola rows. There were 38–46 areolae in 10 µm. The areolae were arranged in transapical rows, with double puncta forming the striae. The valves bent inwards on the external margin side of the costae and rose gently into a convex shape between the costae. The surface appeared wavy. The characteristics of this species are similar to those shown in electron micrographs of *Epithemiaconstricta* W. Smith (You et al. 2009). However, the individuals observed in Lake Akan differed from those in electron micrographs of *E.constricta*, which had radial symmetry, as shown by Lange-Bertalot and Krammer (1987). Therefore, this species was considered to be *E.* sp.

**Proliferative salinity.** This species grew at all salinities tested in this experiment (0–50‰), but it showed the highest growth rate at 20‰ salinity (Fig. 10).

### ﻿*Epithemiaturgida* Kützing

**Figure 9.**
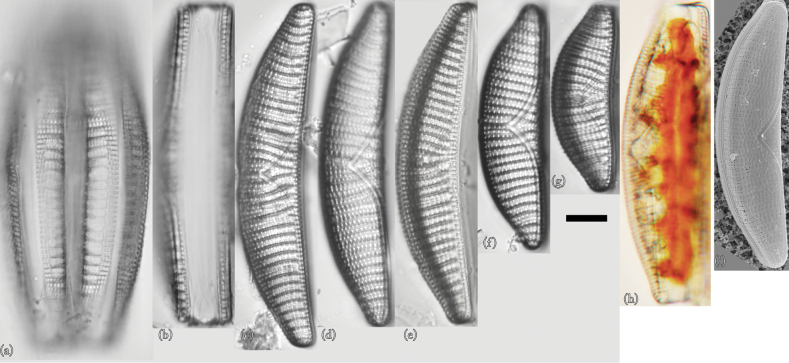
LM (**a–h**) and SEM (**i**) images of *Epithemiaturgida*. Scale bar: 10 μm.

This species is reported by Kawashima and Mayama (2002). Taxonomic examination confirmed that this species is *Epithemiaturgida* (Fig. 9). Individuals observed in culture had universal morphological characteristics recognized worldwide (Krammer and Lange-Bertalot 1988). Many individuals, however, were close to the smallest size reported to date. The central tubercle was located more dorsally than centrally.

**LM morphology.** The valves of this species are similar to those of *E.adnata* and are approximately dorsiventral; the dorsal margin is somewhat convex, the ventral margin is slightly concave, and no protrusion is observed at the apices. Individuals are 48–106 µm in length and 16–18 µm in width (*n* = 50). The biarcuate raphe was located along the ventral border. The central nodule of this species was located more dorsally than centrally (Kawashima and Mayama 2002). There were 8–10 areolae in 10 µm, 9–10 striae in 10 µm, and 4–5 costae in 10 µm, with 2–5 striae between adjacent costae. Costae were near-parallel or radiated slightly.

**SEM morphology.** The appearance of the areola structure was similar to those of *E.adnata* and *E.sorex* (Kawashima and Mayama 2002). Valves showed a very regular arrangement of dome-shaped caps connected at the apical and transapical ends. Two small circular portal veins opened into the cell between costae fibula (You et al. 2009).

**Figure 10.**
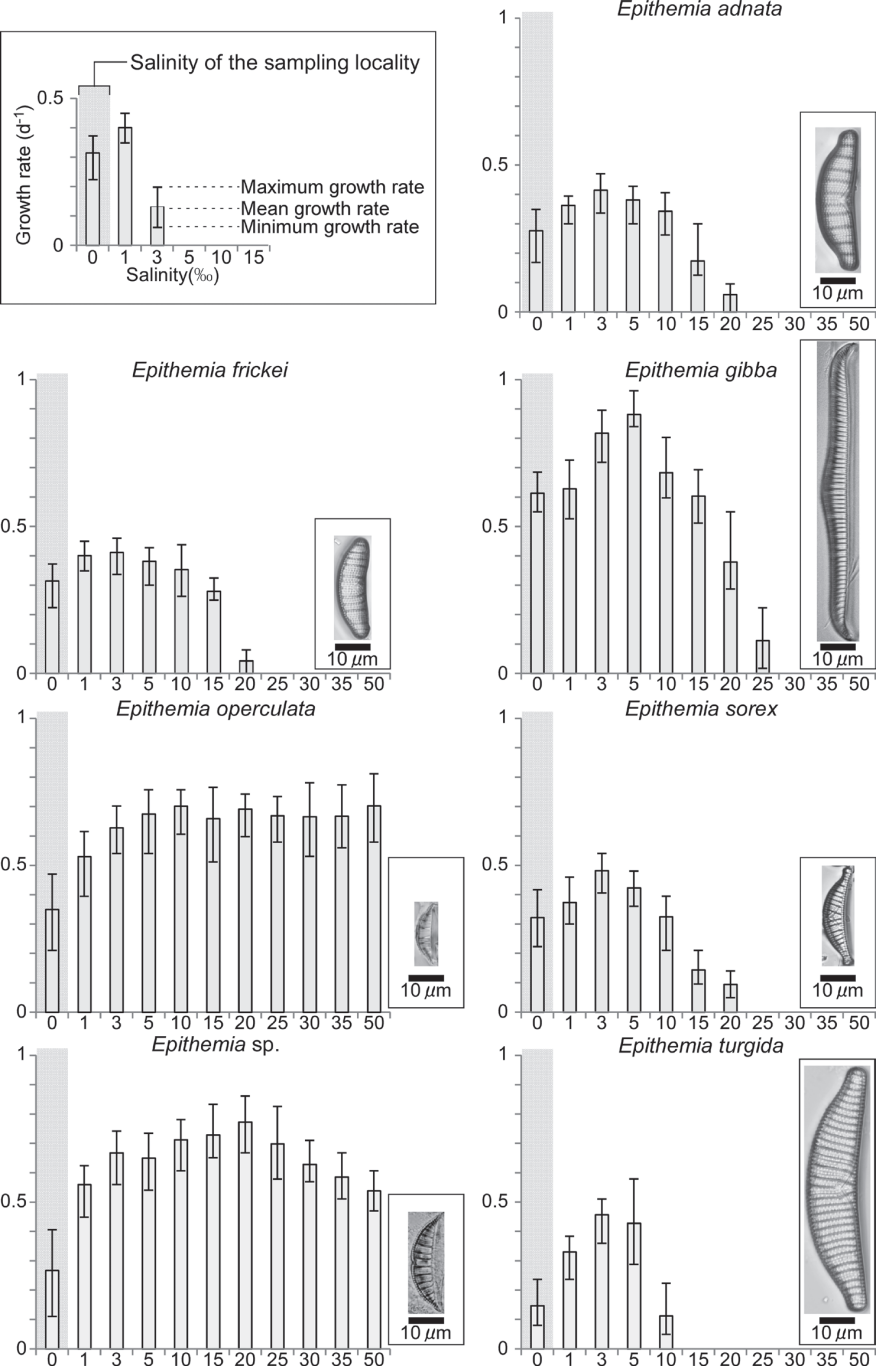
Mean salinity tolerance of the seven *Epithemia* species.

**Proliferative salinity.** This species had the highest growth rate at a salinity of 3‰. There was almost no growth of this species at salinities of 15‰ or higher (Fig. 10).

## ﻿Discussion

The experiments conducted on seven species of *Epithemia* (*E.adnata*, *E.frickei*, *E.gibba*, *E.operculata*, *E.sorex*, *E.* sp. and *E.turgida*) revealed no individual close to the maximum sizes previously described (Krammer and Lange-Bertalot 1988); instead, relatively small ones proliferated. This suggests that these species did not reproduce sexually and did not produce auxospores during the growth period, which may have increased during cell division. However, this observation may have been affected by the limits of the experimental environment; and sexual reproduction may require variations in water temperature and photon flux. The blooming of coastal benthic diatoms may be caused by factors such as water temperature (Admiraal 1977), turbidity (De Seve 1993), and nutrient conditions (Kilroy and Bothwell 2011), which should be further investigated.

*Epithemiaadnata* and *E.frickei* grew between salinities of 0‰ and 15‰ and grew slightly after isolation at salinities ≥20‰ (Fig. 10). The growth rates of these species were the highest in the low-salinity brackish water (3%), which was different from that of the collection site.

*Epithemiagibba* grew slightly after isolation at a salinity of 30‰ but died quickly (Fig. 10). Furthermore, it did not grow at salinities ≥30‰. In contrast, there was growth at a salinity of 0‰, consistent with the classification of Van Dam et al. (1994) and Vos and De Wolf (1993). *Epithemiagibba* is not solely a freshwater species, but it barely grew at salinities ≥25‰, demonstrating its upper limit for salinity tolerance. Although this species is found in freshwater lakes (high electrolytes), our discovery that its optimum salinity was 5‰ classified it as a freshwater to brackish-water rather than a freshwater species.

*Epithemiasorex* grew slightly after isolation at a salinity of 25‰ but died quickly (Fig. 10). Furthermore, it did not grow at salinities ≥25‰. In contrast, growth was observed at a salinity of 0‰, consistent with the findings of Van Dam et al. (1994) and Vos and De Wolf (1993). *Epithemiasorex* is not solely a freshwater species; we found that this species barely grew at salinities ≥25‰, demonstrating its upper limit for salinity tolerance. Although this species is found in freshwater lakes (high electrolytes), our discovery that its optimum salinity was 5‰ classified it as a freshwater to brackish-water rather than a freshwater species.

*Epithemiaturgida* grew slightly after isolation at a salinity of 15‰ but died quickly (Fig. 10). Furthermore, it did not grow at salinities ≥15‰. In contrast, there was growth at a salinity of 0‰, consistent with the findings of Van Dam et al. (1994) and Vos and De Wolf (1993). *Epithemiaturgida* is not solely a freshwater species; our discovery that this species barely grew at salinities ≥10‰ demonstrated its upper limit for salinity tolerance. Although this species is found in freshwater lakes (high electrolytes), our discovery that its optimum salinity was 3‰ or 5‰ classified it as a freshwater to brackish-water rather than a freshwater species.

*Epithemiaoperculata* and *E.* sp. were reported for the first time from Lake Akan in this study. They grew at all salinities tested, even at 50‰; however, *E.* sp. grew faster at higher salinities. These two species were not previously recognised by Kawashima and Mayama (2002), and they appear to be very rare. Although it is not clear why these species were found in Lake Akan, a freshwater lake, they might have been attached to fish transplanted from Lake Abashiri, a northern, brackish lake, to Lake Akan in the 20^th^ century.

The differences between water quality at the collection site (growth environment) and in the culture environment have been demonstrated in a previous study (Yamamoto et al. 2017), which focused on diatom species living on a tidal flat. These species proliferated even in freshwater environments but preferred waters with high concentrations of electrolytes. In a study of diatoms in the Fujimae tidal flat, Yamamoto et al. (2017) reported the ecology of seven diatom species that had a range of salinity tolerance wider than that in the growing environment. The high salinity tolerance of these species contributed to their tolerance to possible drought or rapid temperature increases on the surface of tidal flats caused by tidal changes. In this study, the salinity range tested was not as wide as that experienced by diatoms growing on tidal flats, suggesting that the seven species used in this study may not have been able to grow in moist and rapidly changing environments and are not suitable for tidal flat environments.

There are documented discussions for the reasons for discrepancies between the culture environment and natural habitats (Brand 1984; De Jong and Admiraal 1984; Clavero et al. 2000). Such variations in growth may be due to interspecific competition (Lewin and Mackas 1972; Admiraal 1984; De Jong and Admiraal 1984) or the influence of nutrient levels on community composition (Underwood et al. 1998; Skinner et al. 2006). Alternatively, natural distributions may not accurately reflect salinity tolerance because of the presence of physiologically differentiated races or cryptic species within a species, each with its narrower tolerance and optimum salinity. Recent molecular biological approaches have revealed the existence of cryptic and pseudocryptic species contained in *Pseudonitzschiadelicatissima* (Cleve) Heiden (Orsini et al. 2004; Ruggiero et al. 2015) and *Naviculaphyllepta* Kützing (Crēach et al. 2006; Vanelslander et al. 2009;). Therefore, extrapolation of the behavior of individual clones to natural conditions must be undertaken cautiously. Nevertheless, identifying the intrinsic responses of specific clones to salinity gradients is crucial in providing important information that can help clarify the impact of environmental factors on the ecology of benthic diatoms.

Another important ecological aspect of diatoms is that individual growth rates and species composition of brackish-water diatoms differ depending on their habitats (Yamamoto et al. 2017). Some species prefer high salinity, even though they may be found in brackish water, whereas others prefer low salinity, highlighting the importance of classification and culture experiments of such low-salinity, brackish-water species to provide accurate numerical values ​​for the reconstruction of paleoenvironments in lakes and marine coastal areas. However, relying solely on the abundance trends of these species for determining whether environments were freshwater or brackish-water may lead to incorrect identification of the paleoenvironment. Therefore, to ensure a reasonable interpretation of the paleorecord, it is necessary to understand the production trends of other species and add relevant information. Furthermore, these findings are also important in understanding taphonomic processes (Chiba 2014), such as the effect of the concentration of individual sizes on fossil diatom assemblages.

## ﻿Additional information

### Author ORCIDs

Takashi Chiba https://orcid.org/0000-0001-6431-3645

Yoshifumi Horie https://orcid.org/0000-0002-4558-8624

Akihiro Tuji https://orcid.org/0000-0003-2527-5986

### Data availability

All of the data that support the findings of this study are available in the main text.
